# Icariin: A Promising Natural Product in Biomedicine and Tissue Engineering

**DOI:** 10.3390/jfb14010044

**Published:** 2023-01-12

**Authors:** Zahra Seyedi, Mohammad Sadegh Amiri, Vahideh Mohammadzadeh, Alireza Hashemzadeh, Aliakbar Haddad-Mashadrizeh, Mohammad Mashreghi, Mohsen Qayoomian, Mohammad Reza Hashemzadeh, Jesus Simal-Gandara, Mohammad Ehsan Taghavizadeh Yazdi

**Affiliations:** 1 Department of Stem Cells and Regenerative Medicine, Royesh Stem Cell Biotechnology Institute, Mashhad 9188758156, Iran; 2 Department of Cancer and Oncology, Royesh Stem Cell Biotechnology Institute, Mashhad 9188758156, Iran; 3 Department of Biology, Payame Noor University, Tehran 19395-4697, Iran; 4 Department of Pharmaceutical Nanotechnology, School of Pharmacy, Mashhad University of Medical Sciences, Mashhad 91778, Iran; 5 Industrial Biotechnology Research Group, Institute of Biotechnology, Ferdowsi University of Mashhad, Mashhad 9177948974, Iran; 6 Applied Biomedical Research Center, Mashhad University of Medical Sciences, Mashhad 91778, Iran; 7 Nutrition and Bromatology Group, Department of Analytical and Food Chemistry, Faculty of Food Science and Technology, University of Vigo, Ourense Campus, E32004 Ourense, Spain

**Keywords:** Icariin (ICRN), natural product, cancer therapy, tissue engineering, cell cycle, apoptosis

## Abstract

Among scaffolds used in tissue engineering, natural biomaterials such as plant-based materials show a crucial role in cellular function due to their biocompatibility and chemical indicators. Because of environmentally friendly behavior and safety, green methods are so important in designing scaffolds. A key bioactive flavonoid of the *Epimedium* plant, Icariin (ICRN), has a broad range of applications in improving scaffolds as a constant and non-immunogenic material, and in stimulating the cell growth, differentiation of chondrocytes as well as differentiation of embryonic stem cells towards cardiomyocytes. Moreover, fusion of ICRN into the hydrogel scaffolds or chemical crosslinking can enhance the secretion of the collagen matrix and proteoglycan in bone and cartilage tissue engineering. To scrutinize, in various types of cancer cells, ICRN plays a decisive role through increasing cytochrome c secretion, Bax/Bcl2 ratio, poly (ADP-ribose) polymerase as well as caspase stimulations. Surprisingly, ICRN can induce apoptosis, reduce viability and inhibit proliferation of cancer cells, and repress tumorigenesis as well as metastasis. Moreover, cancer cells no longer grow by halting the cell cycle at two checkpoints, G0/G1 and G2/M, through the inhibition of NF-κB by ICRN. Besides, improving nephrotoxicity occurring due to cisplatin and inhibiting multidrug resistance are the other applications of this biomaterial.

## 1. Introduction

Nowadays, an international movement has engaged targeting the development of natural products for biomedical uses in order to decrease or remove the side effects of non-biological materials [1,2]. A wide-range of naturally occurring materials derived from plants has been tested for their applications in cell toxicity and medicine [3,4,5]. The application of natural materials has been documented in the earliest cultures such as China and Iran, where they relied on plant-derived compounds for health and medical aims and healing purposes [6]. The employment of biomolecules for use in various industries has become an effectual manner in several applications comprising, biocompatible polymers, nano-structural materials, drug delivery, foods and the pharmacological industries [7,8,9].

The growing subject of tissue engineering is very multifaceted and links specialists from varied arenas of materials engineering, mechanical, medicine, and other subjects of bio-sciences [10]. A number of scaffolds prepared by diverse materials have been employed in tissue engineering [9]. Without considering the kind of tissue, several key features such as biocompatibility, mechanical belongings, and used methodology of synthesis are important in scaffold designing [11,12]. Normally, classified groups of materials, such as ceramics and polymers are employed in scaffolds research. Natural biopolymers for instance plant-based materials can show a chief role in cell behavior formation, mostly in regard to chemical indicators and bio-compatibility [13,14,15,16]. However, some factors for example poor mechanical properties and fast biodegradability reduce their efficacy. However, these weaknesses could be bypassed using a crosslinking technique with suitable cross linkers or by a combination of natural polymers. Plants and plant-derived materials are regularly explored for various biomedical uses [17,18,19,20]. Green methods are of special importance in the design of scaffolds due to their high safety and environmental friendliness [21,22]. The uses of nontoxic production technologies and the usage of natural resources can stop pollution and really decrease the uses of unsafe things for the assembly of scaffolds [23,24]. Tissue engineering includes a set of biologically active molecules, engineering and biochemical processes, and the production of materials to make, modify, or treat damaged tissues [25,26,27,28].

ICRN is a kind of flavonoid considered as the key bioactive of the *Epimedium* herb, which has long been utilized in common Chinese medical research. In primary works, the studies on ICRN were generally focused on increasing anti-aging and reproductive activities [29,30]. In current years, with the deepening of medical investigations, positive development on bio-properties of ICRN has been made in immune, cancer, and protective systems [31,32]. So far, various effects of ICRN were proven in osteoporotic bone regeneration [33], attenuates pulmonary fibrosis [34], improvement in chronic kidney issues [35], and many more. Given the beneficial properties of *Epimedium*, this plant has been included into Chinese pharmacopeia [36,37]. Therefore, in this review, the most important biomedical and biomaterials activities of ICRN are taken in to consideration. The following search strategy was conducted. Several relevant literature databases (PubMed, Scopus and Web of Science) were searched. The relevant works were selected using the following keywords in various combinations: Icariin, cell cycle, cancer, apoptosis, and angiogenesis.

## 2. Botanical Origins and Distribution

**Figure 1:**
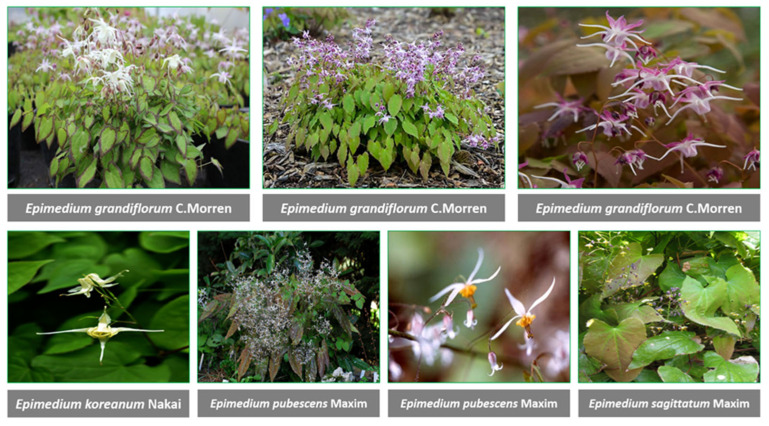
Various species of *Epimedium*.

The genus *Epimedium* L. from Berberidaceae with chief remedial species, comprises a total of 71 species universally [38]. Various species of *Epimedium* have long been recruited in Chinese medication. Among them, the most popular and commercially important are *Epimedium koreanum* Nakai, *Epimedium pubescens* Maxim., *Epimedium brevicornu* Maxim., *Epimedium sagittatum* (Siebold & Zucc.) Maxim. and *Epimedium wushanense* T.S.Ying which is distributed broadly from Japan to Algeria [39,40]. *Epimedium* taxa grows mostly on cliffs under high humidity, and wet lands at height above sea level ranging from 200–3700 m [39]. *Epimedium* is a slow-growing herb with leathery leaves and its stems extend beneath the ground. The leaves are different, lengthy-petiolate, ternately divided twice. The pendant-shaped flowers have lengthy spurs and show a discrepancy in color with colorful flowers including four sepals and four petaloides. It has been shown that a lot of species of this plant (Figure 1) have aphrodisiac assets.

## 3. Traditional Applications and Ethnopharmacology of *Epimedium* Species

Several species of *Epimedium* was used in traditional Asian medicine. In China and Japan, *Epimedium sagittatum* (Sieb. & Zucc.) Maxim. and *Epimedium grandiflorum* have been used to treat impotence, prospermia, hyperdiuresis, osteoporosis, menopause syndrome, rheumatic arthritis, hypertension, and chronic tracheitis [41]. In Korea, Sam-ji-goo-yeop-cho, the herb *Epimedium koreanum*, was traditionally used for impotence, spermatorrhoea and forgetfulness. Now, the major five *Epimedium* species, *Epimedium brevicornum* Maxim, *Epimedium sagittatum* (Sieb. and Zucc.) Maxim., *Epimedium pubescens* Maxim. *Epimedium wushanense* T.S. Ying and *Epimedium koreanum* Nakai are designated as the official sources of *Herba Epimedii* in the Chinese Pharmacopoeia (The State Committee of Pharmacopeia, 2005) [42]. *Herba Epimedii* has a long history as a medicinal plant to treat a wide range of complaints. As far as we know, the earliest record was in Shen Nong Materia Medica which was written in the Eastern Han Dynasty. In this famous medical classic, it was stated that the root and leaves can all be used for treatment, but the details are not very clear. Annotation of Materia Medica is one of the pharmacopoeias published by the government of the Tang Dyansty, but the record is also very simple. The characteristics of *Herba Epimedii* were exactly stated in the Compendium of Materia Medica that was completed by Li Shi-Zhen in the Ming Dyansty. It was stated that *Herba Epimedii* can strengthen bones and muscles, tonify pneuma, reinforce the liver and kidney, enhance psychic energy. The PRC codex also records that *Herba Epimedii* has all these effects and can be used for therapy for some related diseases. Modern pharmacological research displayed that *Herba Epimedii* and its extracts have many kinds of bioactivities that include stimulation of osteoblastogenesis and suppression of the activity of osteoclasts. It inhibits the invasion and migration of cancer cells, improves sexual function, enhances memory, increases the activity of phytoestrogens, promotes the immunological ability and has antiinflammatory properties. So *Herba Epimedii* is always used for the treatment of osteoporosis, tumors, erectile dysfunction, Alzheimer disease and menopausal syndrome in clinical practice [43].

## 4. Physicochemical Properties

**Figure 2:**
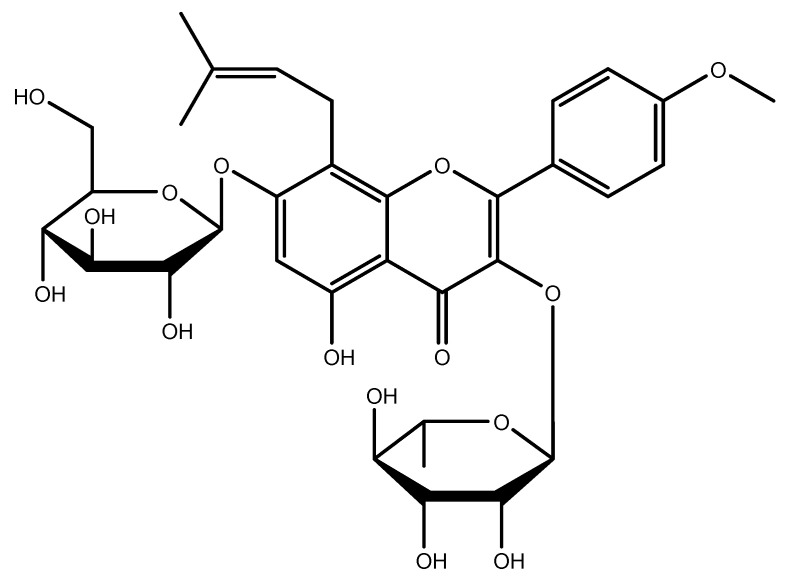
Chemical structure of ICRN.

ICRN (C_33_H_40_O_15_, M_w_ = 676.67) is a type of prenylated flavonoid, showing extensive bioactivities such as antioxidant [44,45,46,47], neuroprotective [48,49,50,51,52], and antitumor [53,54,55,56,57,58,59,60,61] behavior as well as anti-inflammatory responses [62,63,64,65,66,67,68], and can be used to treat erectile dysfunction [69,70,71,72]. It appears it can improve the function of organs including bones and the heart [73,74,75,76,77,78,79,80,81,82,83,84,85,86]. It is crystalline and stable at a low temperature (−20 °C) for approximately two years [87,88,89,90]. The chemical structure is depicted in Figure 2. The stock solutions of ICRN were usually made in DMF or DMSO (20 mg/mL). The solubility of ICRN in organic solvent increases as the temperature increases. To prevent degradation, purging inert gases could be useful. Due to low solubility and stability considerations in water, freshly prepared solutions in the buffer can be used. Usually, a stock solution in DMSO was diluted to increase the solubility in a buffer (the ratio of DMSO to PBS can be 1:10 at pH 7.2) a [87,88,90].

**Figure 3:**
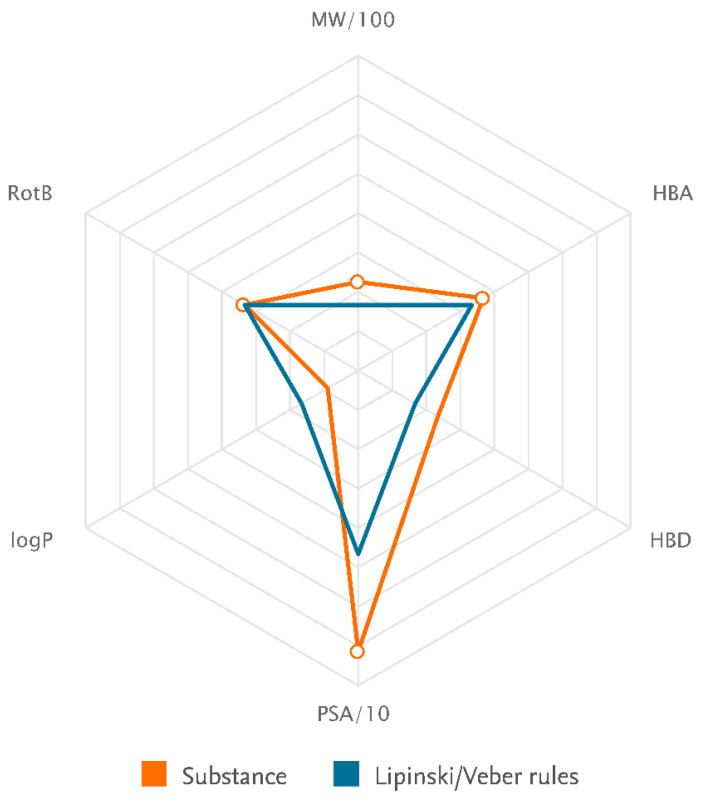
The illustration of the compatibility of substance physicochemical properties with Lipinski/Veber rules component (National Center for Biotechnology Information (2022). PubChem Compound Summary for CID 72302. Retrieved from https://pubchem.ncbi.nlm.nih.gov/compound/72302) (accessed on 1 January 2023).

ICRN is a light yellow-to-yellow powder. It is a flammable solid with a predicted flash point of 300.9 °C and a predicted boiling point of 948.5 °C (at 760 mmHg). It can be stored at temperature. It is partly miscible with water and soluble in pyridine. In a DMSO: PBS (PH = 7.2) ratio of 1:10, the solubility is approximately 0.1 mg/mL. The solubility in DMSO, DMF, and ethanol is 50 mg/mL, ≈20 mg/mL, and <1 mg/mL at 25 °C. In solution, it has no turbidity and the color could be in clear from colorless to dark yellow (Sigma-Aldrich and Santa Cruz safety data sheets). The IUPAC name is 2-(4-methoxyphenyl)-8-(3-methylbut-2-enyl)-7-[(2S,3R,4S,5S,6R)-3,4,5-trihydroxy-6-(hydroxymethyl)oxan-2-yl]oxy-3-[[(2S,3R,4R,5R,6S)-3,4,5-trihydroxy-6-methyloxan-2-yl]methoxy]chromen-4-one, which was computed by LexiChem 2.6.6. To the best of our knowledge, the chemical, physical, and toxicological properties have not been thoroughly explored. The known calculated data for the drug likeness of icariin were shown by Lipinski, the rules and Veber rules components, which revealed only one matched criterion in both cases (Table 1, Figure 3). According to the rules of Lipinski, the hydrogen bond donor (HBD), hydrogen bond acceptors (HBA) and octanol-water partition coefficient (log P) should be less than or equal to 5, 10, and 5, respectively. The molecular weight must be less the 500 [91]. Therefore, logP is only acceptable according to the Lipinski rules component. According to Veber’s rule, bioavailability is acceptable only when less than ten rotatable bonds (RB) exist in the molecular structure and the polar surface area (PSA) was less than 140 [92]. Icariin can be metabolized into nearly 10 constituents. The icaritin and icariside II are the metabolized forms of icariin in the small intestine, showing lipid-lowering and lipid-regulating effects. Icariside II can be absorbed in the bloodstream [93]. In many physiological complex systems, icariin could also improve bioactivity and biocompatibility [94,95,96].

In UV-visible spectroscopy, the absorption peak of 268 nm can be seen in the curve. In the FTIR spectrogram, the characteristic peaks at ≈1650, 1600, 1510, 1070, and 1260 cm^−1^ can be assigned to free icariin. In differential scanning calorimetry, the endothermic peak at ≈250 °C is related to icariin [97].

## 5. Bioengineering Application of ICRN

### 5.1. Bone Tissue Engineering

Bone disorders are becoming usual frequently as the aging community grows, and bone breaks often moreover happen. The treatment of major bone defects remains a main challenge [98]. The necessity for osteogenic bone alternatives causes the advancement of approaches in bone material engineering. A usual material engineering plan typically contains cells, biomaterials, and bioactive scaffolds [99]. Autologous multipotent cells have been broadly employed for bone renewal [100]. Though a perfect bone tissue engineering scaffold must be both osteoconductive and osteoinductive [101], most polymers and metal scaffolds are only osteoconductive [102,103]. To increase the beneficial properties, biological factors are generally used by viral gene or protein delivery [104,105]. Even though an employment of bone morphogenetic proteins (BMPs) has been widely considered for bone renewal, huge volumes of BMPs are essential and BMP-comprising plans tend to fail in a certain proportion of cases, thus raising worries over expenses and safety [106,107]. The high cost and quick ruin of BMPs and other protein drugs [108] bound their experimental usage, too. Hence, there is a critical need for alternative agents with higher usefulness and cost-effectiveness. There is a limited amount of information on low molecular weight agents that effectually enhance bone construction, for instance statins [109] and isoflavone products [110]. With the advances in separation procedures, it has become more facile to acquire purified molecules such as ICRN from natural sources instead of using unsafe materials, which is even expensive.

In a study, ICRN encapsulated with PLGA was implanted in 3-dimensional printed polycaprolactone/nano-hydroxyapatite scaffolds to simplify in-situ regeneration of bones. Such a scaffold displayed outstanding mechanical efficiency due to the nano-hydroxyapatite and revealed sustainable diffusion of ICRN as the polycaprolactone degraded [111]. Another study represented that ICRN can activate both ERα (non-genomic estrogen receptor) and Akt (serine/threonine-specific protein kinase) by increasing rapid induction of insulin-like growth factor I (IGF-1) signaling in osteoblastic cells for osteogenesis [112]. It is demonstrated that ICRN promotes and inhibits, respectively, humanoid osteogenic and adipogenic differentiation of bone marrow mesenchymal stem (BM-MS) cells via activating of the Wnt/β-catenin pathway mediated by microRNA 23a [113]. In a work, the released drug from thiolated ICRN/biphasic calcium phosphate scaffolds could increase the migration, proliferation and osteogenesis of ovariectomized rat BM-MS cells, and upregulate the angiogenic gene expression in humanoid umbilical vein endothelial cells *in vitro* [33]. ICRN in combination with other substances can be even more effective. A study by Don et al. showed that fetal bovine serum exosomes-ICRN more efficiently increased the osteoblasts’ proliferation than the time ICRN was used alone [114]. It was shown that ICRN is considered a factor for knee osteoarthritis by involving four pain-related genes as well [115].

### 5.2. Cartilage Tissue Engineering

Osteoarthritis is the most public chronic joint sickness and is related with signs for instance, cartilage damage and deprivation. Cartilage displays too low affinity to self-repair because of the little regenerative ability of resident chondrocytes and the privation of vascular, nervous, and lymphatic structures [116]. Existing clinically presented cartilage treatments consist of autologous chondrocyte implantation, matrix induced autologous chondrocyte implantation, microfracture, and abrasion arthroplasty. On the other hand, these plans are only responsible for sub-optimal clinical results and are frequently not enough for keeping the durable function of articular cartilage [117]. Because of these problems, studies in recent years were intensive on cartilage engineering which cells, functional molecules, and scaffolds to lead cartilage creation. Chondrocytes are the best cell resources for cartilage tissue treatment. Nevertheless, chondrocytes have a tendency to miss their phenotype and go through hypertrophy upon *in vitro* growth, that could be described by reduced fabrication of proteoglycan and type II collagen, and enhanced secretion of type I and type X collagens [118]. Hence, existing approaches for applied material engineering scaffolds for preserving the differentiated state of chondrocytes, endorsing cartilage creation, and the latest works revealed that a suitable scaffold with organized release biomolecules may perhaps be more in effect [119,120]. Numerous works also revealed that ICRN is able to enable the chondrogenesis of mesenchymal stem (MS) cells in an accustomed medium, provide propagation of chondrocytes, keep the phenotype of chondrocytes, stimulate the secretion of proteoglycan and the collagen matrix and prevent the deprivation of collagen and proteoglycan [121,122]. Furthermore, in cooperation with exogenous growth regulators, ICRN is low-cost, constant and non-immunogenic [123]. As a result, ICRN has been widely employed in scaffolds to increase speed tissue regeneration [124]. Some works proposed that an upper concentration of ICRN can motivate chondrocytes to secrete a further cartilage matrix within the in effect harmless concentration variety [125]. It was indicated that ICRN could prevent cell propagation at concentrations above 10 mM [126].

With the aim of enhancing the ICRN loading to retain the enduring biological property of the scaffolds, ICRN was fused into the hydrogel scaffolds or chemical crosslinking. The achieved hydrogel scaffolds continued an elongated smooth release of drugs and permitted the encapsulated chondrocytes to secrete more proteoglycan and the collagen matrix, but its poisonousness to the encapsulated cells is not adopted for clinical uses [123,127].Some bioengineering applications of ICRN are listed in Table 2.

## 6. Cancer Therapy by ICRN

### 6.1. Apoptosis Induction

**Figure 4:**
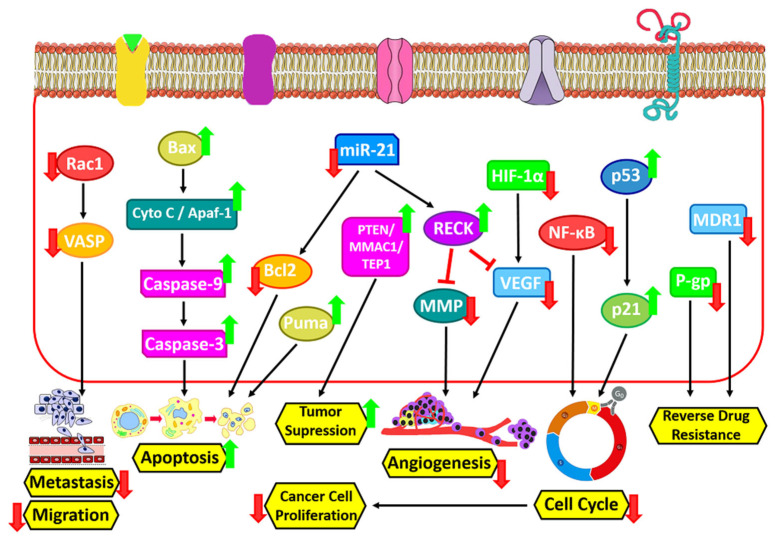
Cancer therapy by ICRN.

The planned cell death that occurs in the organs of the body is called apoptosis. Biochemical changes including mixing, nuclear disintegration, chromatin density, DNA cleavage, mRNA decay, and eventually cell shrinkage alter cell morphology and other characteristics and cause cell death [131]. ICRN and its derivatives exert their apoptotic effect by activating the innate pathway of apoptosis. ICRN and icaritin increase cytochrome c secretion, Bax/Bcl2 ratio, poly (ADP-ribose) polymerase and caspase stimulation in various types of cancer cells (Figure 4) [132]. MiR-21 is extremely expressed in numerous human tumors and can cause cell proliferation, differentiation and apoptosis, and also play a key role in the formation of metastases in tumors. In a study by Li et al., they showed that ICRN can stimulate caspase-3 activity, block the expression of miR-21, and induce cellular apoptosis in ovarian cancerous cells. Wang et al. represented that ICRN inhibits Bcl-2 protein expression and induces apoptosis in MLTC-1 cells by regulating Bcl-2/Bax expression (Figure 4) [133]. In cancer cells, ICRN significantly reduces the expression of microRNA 21 and the Bcl-2 protein, and increases the expression of PTEN and RECK protein (Figure 3) [134]. According to Sharma et al., however, ICRN treatment noticeably leads to CDK2, CDK4, Cyclin D1, Bcl-2, and Beclin-1 downregulation, it up-regulates the expression ranks of caspase 3 (Figure 4), PARP and p62 [135].

The results of Western blot tests by Fan et al. to evaluate the anti-cancer action of ICRN in esophageal squamous cells carcinoma (ESCC) showed that ICRN upregulated the expression of GRP78 ATF4 and CHOP. It also upregulated p-PERK and p-eIF2α amounts in EC109 and TE1 cells in an amount-dependent way as well as increased Puma expression and decreased Bcl2 expression. In this study, the apoptotic index of ESCC was evaluated after concentrations of 20, 40 and 80 μM ICRN for 24 h. The results showed an enhancement in the stimulation of apoptosis with an increasing ICRN dose [136]. Moreover, ICRN induces the apoptosis process through the upregulation of Bax/Bcl-2 and induce ROS in a mitochondria mediated way. Notably, the blockage of breast cancer cells invasion through the abating of NF-κB/EMT activation by ICRN as shown [137]. In another study it was reported that ICRN inhibits the proliferation and migration of ovarian cancer cells *in vitro* and stimulates apoptosis via stopping the PI3K/AKT signaling [138].

### 6.2. Inhibition of Cancer Cell Proliferation

ICRN stops the cell cycle at the G0/G1 and G2/M phases by inhibiting NF-κB activity and thus blocks the growth of cancer cells (Figure 4) [132]. Song et al. reported that *in vitro*, ICRN induces dose/time dependent cell toxicity to MDA-MB-231, MDA-MB-453, and 4T1 cell lines, and inhibits breast cancer cell proliferation [137]. They also used colony formation methods to confirm the anti-proliferative effect of ICRN. The ability to form colonies of cells concentrated with 10 or 20 μM ICRN was considerably repressed and the size of colonies uncovered to ICRN was reduced in comparison to the control group. ICRN can inhibit the tumor growing *in vivo*. To see if the anti-tumor activity of ICRN *in vivo* is similar to its effects *in vitro*, tumor-bearing mice MDA-MB-231 and 4T1 were treated with doses of 20 and 40 mg/kg of ICRN. A therapeutic dosage of 20 mg/kg (*p* < 0.01) and therapeutic quantity of 40 mg/kg (*p* < 0.005) significantly inhibited the growth of the MDA-MB-231 tumor in a dose-dependent way compared to the control group. The mice did not show any abnormal alterations in body weight during treatment. Immunohistochemical results of tumors showed that ICRN inhibits the proliferation of Ki-67-positive cells, increases caspase-3 expression, induces cell apoptosis, and regulates NF-κB expression. Moreover, Western blot results of tumors indicated that ICRN increases the SIRT6 expression level, inhibits NF-κB p65 nuclear expression, and decreases PD-L1 expression. ICRN was similar in the model of 4T1 tumor-bearing mice, and was able to reduce tumor cell proliferation and NF-κB expression levels and stop tumor growth (Figure 4) [137]. A latest work by Wang et al. on the therapeutic properties of ICRN at concentrations of 25 μM, 50 μM, and 100 μM for 48 h in relation to cancer showed that this biomaterial could be significantly dose-dependent and inhibit SKOV3 cell proliferation [138].

### 6.3. Angiogenesis Inhibition

ICRN stops the proliferation, migration and formation of cancer cells. Moreover, it has anti-angiogenic action *in vivo* in various tumor models. CD31 is a pan-endothelial indicator and is expressed on the surface of endothelial cells. ICRN treatment significantly reduced CD31-positive regions in tumor mice. As is clear, the vascular endothelial growth factor (VEGF) is an important growing agent that acts as a major controller of angiogenesis. The consequences of both *in vitro* and *in vivo* investigation show that the decrease in VEGF levels by down regulation of this factor in both ICRN treated groups showed the anti-angiogenic effect of this biomaterial (Figure 4).

Hypoxia-inducible factor-1α (HIF-1α) is a substantial goal in solid tumors therapy and is induced by hypoxia in a dose-dependent way in cancer cells. It is assumed that Icariside II (one of the ICRN-derivatives) increases the interaction between HIF-1α and von Hippel-Lindau (VHL) whereby a decrease in the protein level of HIF-1α occurs (Figure 4) [139]. Additionally, Icariside-II blocks the migration rate in human osteosarcoma cells and the tube creation rate in humanoid umbilical vein endothelial cells.

RECK is a recently explored tumor-inhibitor gene and is known as a matrix metalloproteinase inhibitor, in turn, decreasing the tumor invasion and angiogenesis [140]. The influence of ICRN on the expression of the RECK protein in A2780 ovarian cancer cells was tested. The consequences displayed that treatment with ICRN for 2 days at dose of 25 and 50 μM significantly enhanced the expression level of the RECK protein compared to the control group (Figure 4) [134].

### 6.4. Metastasis and Migration Inhibition

Metastasis is the movement of a pathogen from a primary site to a secondary site in the host body. That is very common in the later stages of cancer and occurs through the blood, lymph, or both. The most common sites of metastasis are the lungs, liver, brain, and bones. The anti-metastatic activity of ICRN has also been investigated. A study of very metastatic human lung cancerous cells after treatment with ICRN displayed a reduction in the capability of these cells to attack and migrate (Figure 4) [141]. Another study on ICRN indicated that it could suppress the adhesion of lung adenocarcinoma by acting on vasodilator phosphoprotein (VASP), which is important in cell migration along with tumor metastasis. This study also showed that this biomaterial inhibits gastric cancerous cell invasion and cell migration and is the main process for suppressing the expression of genes associated with Rac1 and VASP cell motility (Figure 4) [142]. The results of these works showed that ICRN and its derivatives prevent cancer cell metastasis by regulating proteins that are critical for cancer metastasis [143,144]. A study by Song et al. found that ICRN could inhibit the pulmonary metastasis pattern of 4T1 cells in BALB/c mice. Moreover, lung tissues were examined using H&E staining to estimate the anti-metastatic efficiency of ICRN. In these tissue sections, H&E staining showed that there were fewer tumor nodules in the ICRN group compared to the control group [137].

### 6.5. Regulation of Immune System

Immunomodulation is thought to revolutionize the treatment of inflammation-related diseases such as autoimmune diseases, and cancer. [145]. The immune system in these situations could be either motivated or inhibited by biomaterials. ICRN has been shown to reduce tumor progress and the ratio of Myeloid-derived suppressor cells (MDSCs) [132]. Liu et al. reported the sex hormone-like properties of ICRN on T-cells immune modulation in spontaneously hypertensive rats. Their results proved that ICRN can regulate T-cells differentiation related to blood pressure reduction in SHR rats [146]. According to these studies, the effect of modulating the immune system of ICRN causes anti-tumor immunity in killing tumor cells and stunting tumor cells.

In another study, due to the effective inhibitory result of ICRN on the NF-kB signaling way, the ability of ICRN to suppress the microenvironment of the tumor environment was investigated. MDSCs, which typically accumulate in tumors, are responsible for suppressing the immune system’s microenvironment. In the study of the effect of ICRN on CD4+ and CD8+ T-cells, the results represented that the ratio of tumor infiltration to CD4+ and CD8+ cells was meaningfully enhanced. In addition, it significantly regulated the proportion of MDSCs in the tumor compared to the control group. In general, ICRN can improve the microenvironment of the tumor suppressor immune system, thus increasing the anti-tumor effect [137].

## 7. Effects of ICRN on Drugs Used in Cancer Therapy

### 7.1. Multidrug Resistance (MDR) Inhibition

Multidrug Multidrug resistance is a principal concern in community health. It defines a complex phenotype whose principal feature is resistance to a wide-ranging of structurally unrelated cell toxicity complexes, many of which are anticancer factors [147]. Moreover, MDR could be caused by a species of micro-organism to at least one antimicrobial drug in three or more antimicrobial classes [22,24,148]. Some studies were performed to overcome drug resistance induced by tumor cells with ICRN and its derivatives. A study on the adriamycin-resistant ADR-resistant human hepatocellular cell line found that ICRN-derived icarithin can reduce ADR cytotoxicity by decreasing MDR1 gene expression, decreasing P-gp levels, and increasing intracellular accumulation ADR [149]. PTEN gene deficiency is present in malignant tumors and tumor cell lines (Figure 4) [150]. Lee et al. showed that ICRN enhances PTEN protein expression in ovarian cancer cells [134].

### 7.2. Effects of ICRN on Cisplatin

ICRN, as a major and remarkable flavonoid is utilized in cancer therapy. Cisplatin, as an efficient chemo-remedial factor, is usually employed to treat several kinds of cancers [151,152]. Nephrotoxicity stimulated by cisplatin extremely limits its clinical uses [153,154,155].

**Figure 5:**
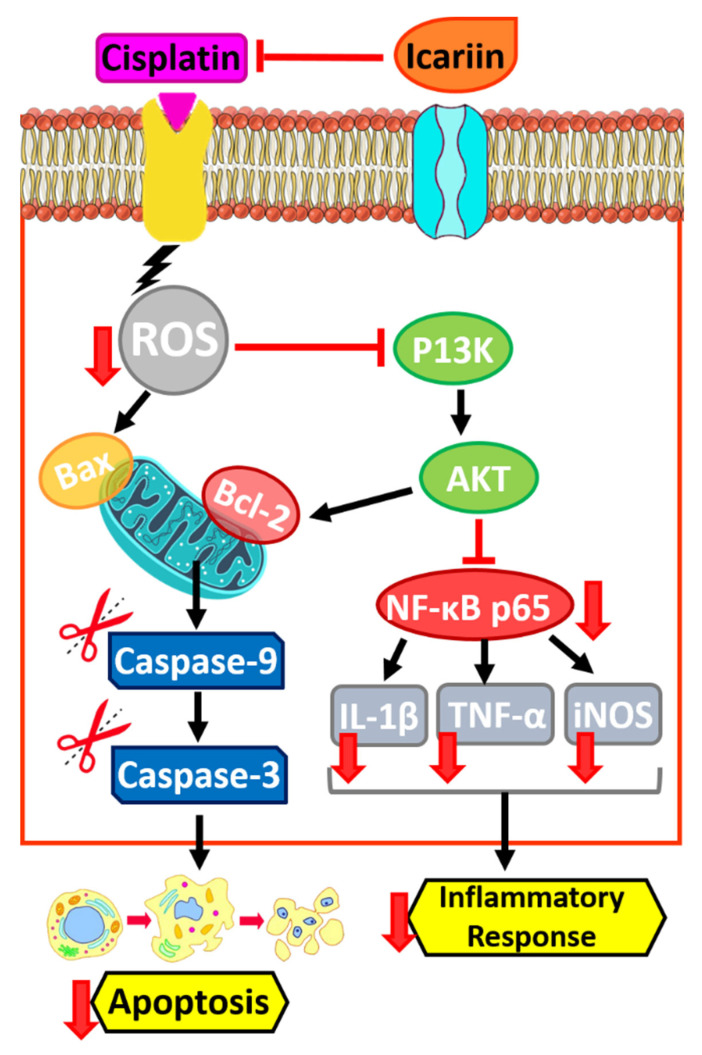
Effects of ICRN on Cisplatin in cancer therapy.

The mechanism principal for the decrease in cisplatin-induced renal damage using ICRN was investigated. Treatment of mice with cisplatin cause renal injury, displaying enhancement in blood urea nitrogen, tubular damage, and apoptosis. These renal alterations could be meaningfully improved by ICRN. The results show that ICRN decreased the expression of TNF-α and NF-κB, cleaved caspase-3, and Bax, as well as enhanced the expression of BCL-2 [156]. It is reported that ICRN increases the chemosensitivity of cisplatin-resistant ovarian cancerous cells by inhibiting autophagy using activation of the AKT/mTOR/ATG5 way (Figure 5) [157].

## 8. Other pharmacological Effects of ICRN

Natural products have contributed to the improvement of numerous drugs for various signs. It is becoming clear that many natural products are able to influence receptor positions on or inside cells, just as a drug must do [158]. Natural-product frameworks are labelled as privileged constructions. Several *in vivo* works verified the useful result of ICRN on reproductive utilities. Treatment of ICRN meaningfully enhances epididymal sperm counts and testosterone ranks of male rats [71]. ICRN as well effectively enhanced the erectile action in gelded wistar rats using an enhancing proportion of smooth muscle and inducible NO-synthase in the corpus cavernosum [159]. ICRN is a main biological pharmaceutical material with strong cardiovascular protecting roles [160]. Emerging data in the previous reports revealed that it has numerous atheroprotective roles, by several mechanisms, comprising decreasing DNA destruction [161], modifying endothelial dysfunction [162], preventing the propagation and migration of smooth muscle cells [163], inhibiting macrophage derived foam cell creation and inflammatory responses. [164,165].

Two main hallmarks are regarded as Alzheimer’s disease (AD): extracellular gathering of amyloid b peptide (Ab) and intraneuronal accumulation of the tau protein as well-known as neurofibrillary tangles (NFTs) [166]. Guo et al. proposed that ICRN can progress spatial learning and memory capabilities in rats with brain dysfunction prompted by lipopolysaccharide (LPS), a result which could be caused by reduced expressions of TNFα, IL-1β and COX2 in the hippocampus (Table 3) [167].

## 9. Conclusions

Use of bio-scaffolds in tissue engineering is an inevitable application, but biodegradability and biocompatibility are the main properties of scaffolds that should be considered. Animal or plant derived proteins that are recognized as natural biopolymers have critical impacts on cell behaviors. Interestingly, safer and eco-friendly greener technologies resulting from plant-based polymers can make promising effects on regenerative medicine and tissue engineering. ICRN as a reliable plant biomaterial shows many effects in medical treatment such as increasing osteogenesis by induction of IGF-1, also by activating the Wnt/β-catenin pathway and inhibiting adipogenesis. Preventing cell propagation at concentrations above 10 mM, inducing the apoptosis process through the upregulation of Bax/Bcl-2, inducing cell apoptosis due to increasing caspase-3 expression, suppressing the adhesion of lung adenocarcinoma by acting on VASP thereby influencing cell migration as well as tumor metastasis are the other applications of ICRN. Besides, the elongated smooth release of drugs giving permission to the encapsulated chondrocytes to secrete more collagen matrix exemplifies the role of ICRN in bio- and tissue engineering when it is combined with hydrogel scaffolds. Tumor progression is often linked with a phenotypic switch from M1 to M2 in tumor associated macrophages (TAMs) in the tumor microenvironment. Seyedi and her colleagues showed that downregulation of STAT3 resulted in the switching of M2 to M1 in the tumor microenvironment, in turn causing inhibition of tumor progression [183]. Moreover, other studies demonstrate the activation of cancer stem cells by increasing IL6 and MFG-E8 secretion stemming from overexpression of STAT3 in the tumor microenvironment [184,185]. Hence, the evaluation of ICRN on STAT3 downregulation in order to switch M2 to M1 in the tumor microenvironment as well as in cancer stemming cell inactivation can be a promising study in the future. Above all, an emphasis is placed on the mechanism of ICRN and its future perspective, aiming at providing a relative theoretical base for the application of ICA in the future biomedical treatment plans.

## Figures and Tables

**Table 1:** Identification and drug likeness of icariin.

Identification	
Chemical Names	Icariin
Molecular Formula	C_34_H_42_O_14_
Lipinski rules component	
Molecular Weight	674.699
logP	2.682
Hydrogen bond acceptors (HBA)	11
Hydrogen bond donors (HBD)	7
Matching Lipinski Rules	1
Veber rules component	
Polar Surface Area (PSA)	214.06
Rotatable Bond (RotB)	10
Matching Veber Rules	0

**Table 2:** Bioengineering application of ICRN.

Scope	Applications	Ref.
Bone tissue engineering	increases rapid induction of IGF-1 signaling in osteoblastic cells and activate both ERα and Akt in osteogenesis promotes the osteogenic differentiation of MC3T3-E1 promotes the healing of calvaria bone in composite scaffold of poly (lactic-co-glycolic acid) (PLGA) microsphere PLGAμs	[112]
	activates Wnt/β-catenin pathway mediated by microRNA 23a and thereby osteogenic differentiation of BM-MSCs	[113]
	increases the proliferation of osteoblasts especially when it is combined with fetal bovine serum exosomes induces significantly the osteogenic differentiation of bone marrow mesenchymal stem cells (BMSCs) by upregulating alkaline phosphatase activity and the expression of bone sialoprotein II (BSPII) and runt-related transcription factor-2 (Runx-2)	[114]
	it is used as a factor for knee osteoarthritis by involving four pain-related genes as well	[115]
	upregulates the expression of osteogenic genes in BMSCs enhances the osteogenesis *in vivo* through the delivery of ICRN by calcium phosphate cement (CPC) scaffolds promotes bone defect repair by anti-osteoporotic effect through the systemic administration	[128]
Cartilage tissue engineering	enables the chondrogenic differentiation of MSCs in an accustomed medium induces the propagation of chondrocytes and keeps the phenotype of chondrocytes stimulates proteoglycan and collagen matrix secretion prevents the deprivation of collagen and proteoglycan *Thiolated icariin (ICRN-SH):* facilitates chondrocyte proliferation maintains chondrocyte phenotype promotes the secretion of the cartilage extracellular matrix	[122]
	Compared with hyaluronic acid/collagen (HA/Col) hydrogel, ICRN-HA/Col hydrogel: upregulates remarkably the chondrogenic genes expression enhances the matrix synthesis of sGAG and type II collagen increases the amount of type II collagen in the neo-cartilage layer *in vivo* upregulates the osteogenic genes, including RUNX2, ALP and OCN at early stage makes more calcium deposition deposited more abundant type I collagen in the newly formed subchondral bone increases the gene expression and matrix synthesis of type X collagen	[123]
	1 × 10^−6^ M concentration of ICRN in differentiation medium of BMSCs enhances cartilage extracellular matrix synthesis increases significantly the gene expression levels of collagen II and SOX9 promotes more chondrocyte-like rounded morphology in BMSCs inhibits the side effect of growth factor activity by preventing further hypertrophic differentiation	[129]
	In the model of osteoarthritis (OA) injected by HA/Poloxamer 407/ICRN hydrogel: promotes the proliferation and chondrogenesis of BMSCs through the Wnt/β-catenin signaling pathway prevents cartilage destruction by stimulating chondrogenic differentiation of BMSCs regulates the expression of cytokines such as IL-10 thereby relieving pain	[130]
	motivates chondrocytes to secrete further cartilage matrix within the in effect harmless concentration variety	[125]

**Table 3:** Other pharmacological effects of ICRN.

Scope	Result	Ref
Neuroprotective	Enhancing mild hypothermia-stimulated neuro shielding Preventing the action of NF-κB in experiential ischemic stroke	[168]
	Reversing the (hypoxic-ischemic) HI-induced decrease in phosphorylated Akt Activation of cleaved caspase3	[169]
	Debilitate amyloid-β (Aβ)-induced neuronal insulin resistance by PTEN down-regulation	[170]
Cardiovascular diseases	Alleviated the severity of diabetes The blood glucose ranks were decreased Serum adiponectin ranks were not changed The mRNA and protein expression rank of p-AMPK and GLUT-4 proteins were enhanced in the T2DM rats treated with ICRN	[171]
	Insulin resistance, left ventricular dysfunction, aberrant lipids deposition, cardiac inflammation and fibrosis were ameliorated by the treatment of ICRN The ranks of extracellular matrix proteins of heart tissue considerably refused	[172]
	Preventing isoprenaline-induced Takotsubo syndrome-like cardiac dysfunction in rats. Reduced the ROS ranks and enhanced antioxidant element expression while decreasing pro-inflammatory factor secretion Inhibiting TLR4/NF-κB signaling path protein expression	[173]
Reproductive system	Preserved ovarian granulosa cells from d-galactose stimulated aging Cell viability enhanced and endogenous β-galactosidase property lower Promoting DNA damage repair	[174]
	Improved the decrease in sperm density, hormone ranks and activities of antioxidant enzymes altered in the nicotine treated mice	[175]
	Improve follicular development; prevent follicular atresia, thus reducing the ovarian hypofunction FSH and LH levels decrease and E2 enhance AMH and Bcl-2/Bax expression up-regulates in aging female mice Partly restore ovarian function of aging mice and increase their fecundity	[176]
Anti-tumor	Inhibition proliferation and metastasis Increases antitumor immunity in triple-negative breast cancer Inhibitory effect on SIRT6/NF-κB/epithelial-mesenchymal transition (EMT) signaling pathway	[177]
	Induces apoptosis of human lung adenocarcinoma cells in a time and concentration-dependent way *in vitro* Activating the mitochondrial apoptotic pathway Activation of members of the caspase family of proteins	[178]
	Tumor necrosis agent-associated apoptosis-stimulating ligand plus ICRN synergistically stimulated apoptosis in 49% of HCT116 colon cancer cells Upregulation of cell surface death receptors Enhancing the production of ROS	[179]
Anti-inflammation	The expression of TDP-43 in chondrocytes reduced Inhibition the elevation of inflammatory cytokines affected by TDP-43 Prevents the JNK and p38 MAPK signaling way in-vitro	[180]
	Promotion of chondrocyte vitality and extracellular matrix synthesis Inhibition of the expressions of NF-κB and HIF-2α in bone defect mice Neutralized the activation of IKK (IKK phosphorylation), the phosphorylation of IkB and NF-κB and the expression of HIF-2α Inhibition of the nucleus transfer of NF-κB and the expressions of MMP9 and ADAMTS5	[181]
	The pathological alteration, collagen deposition could be significantly reversed by ICRN treatment Significantly reduced the protein expression of proinflammatory agents such as nuclear factor-κB, cyclooxygenase-2, interleukin 1-β and prooxidative enzyme (NADPH oxidase-4) Increasing the protein expression of antioxidative enzymes (SOD and CAT)	[182]

## Data Availability

The data availability statement is not applicable to this study.
